# The effect of temperature on the stability of PCSK-9 monoclonal antibody: an experimental study

**DOI:** 10.1186/s12944-021-01447-3

**Published:** 2021-02-25

**Authors:** Tanawan Kongmalai, Nalinee Chuanchaiyakul, Chattip Sripatumtong, Tunsuda Tansit, Yuttana Srinoulprasert, Nareerak Klinsukon, Nuntakorn Thongtang

**Affiliations:** 1 Division of Endocrinology and Metabolism, Department of Medicine, Faculty of Medicine Siriraj Hospital, Mahidol University, 2 Wanglang Road, Bangkoknoi, Bangkok, 10700 Thailand; 2 Department of Immunology, Faculty of Medicine Siriraj Hospital, Mahidol University, Bangkok, Thailand; 3 Division of Endocrinology and Metabolism, Phyathai Hospital, Bangkok, Thailand

**Keywords:** PCSK9 monoclonal antibody, Temperature, Drug stability

## Abstract

**Background:**

PCSK9 monoclonal antibody lowers plasma PCSK9 and LDL-cholesterol levels. The manufacturers recommend drug storage at 2–8 °C, and not above 25 °C. This study aimed to investigate drug stability at various temperatures that this drug could be exposed to during medication handling and transportation in tropical countries.

**Methods:**

Alirocumab and evolocumab were tested in 3 study conditions: room temperature (RT), cooler device with cold pack, and freeze-thaw for 9 and 18 h. Heated drugs were used as negative control. Free plasma PCSK9 levels from 9 hyperlipidemia subjects were measured with ELISA.

**Results:**

Average subject age was 49.2 ± 18.4 years. Percent PCSK9 inhibition significantly declined in heated drugs compared to baseline. Average RT during the study period was 30.4 ±2.6 °C. Change in percent PCSK9 inhibition of PCSK9 mAb at RT from baseline was − 5.8 ± 4.4% (*P* = 0.005) and − 11.0 ± 8.9% (*P* = 0.006) for alirocumab at 9 h and 18 h, and − 9.7 ± 11.8% (*P* = 0.04) and − 15.1 ± 14.3% (*P* = 0.01) for evolocumab at 9 and 18 h, respectively. In contrast, there were no significant changes in percent PCSK9 inhibition from baseline when PCSK9 mAb was stored in a cooler. In freeze-thaw condition, changes in percent PCSK9 inhibition from baseline to 9 and 18 h were − 5.2 ± 2.9% (*P* = 0.001) and − 2.6 ± 4.9% (*P* = 0.16) for alirocumab, and − 1.8 ± 4.2% (*P* = 0.24) and 0.4 ± 6.1% (*P* = 0.83) for evolocumab.

**Conclusion:**

Proper drug storage according to manufacturer’s recommendation is essential. Drug storage at RT in tropical climate for longer than 9 h significantly decreased drug efficacy; however, storage in a cooler device with cold pack for up to 18 h is safe.

**Supplementary Information:**

The online version contains supplementary material available at 10.1186/s12944-021-01447-3.

## Background

Dyslipidemia is a major modifiable risk factor for atherosclerosis, which is a common cause of cardiovascular disease. There is strong evidence that elevated plasma low-density lipoprotein cholesterol (LDL-C) level has a causal relationship with risk of developing atherosclerotic cardiovascular disease (ASCVD) [1]. Proprotein convertase subtilisin/kexin type 9 (PCSK-9) binds to low-density lipoprotein receptor (LDL-R) in hepatocytes and increases endosomal and lysosomal degradation of LDLR [2]. PCSK9 monoclonal antibody (PCSK9 mAb) lowers plasma PCSK9 levels, which prevents LDLR degradation and increases the number of LDLR available to lower low-density lipoprotein in plasma [3]. Previous studies demonstrated the efficacy of PCSK9 mAb to reduce plasma LDL-C levels by 54.7–57.0% when compared with placebo [4, 5]. In addition, PCSK9 mAb achieved plasma LDL-C reductions of 49.8% in patients with high cardiovascular risk, which was significantly greater than the reduction realized from ezetimibe treatment [6]. Moreover, PCSK9 mAb demonstrated efficacy in reducing the incidence of major cardiovascular events (MACE) when added to statin therapy [7, 8]. Two PCSK9 monoclonal antibodies, alirocumab and evolocumab, are currently commercially available. These drugs are indicated as an adjunct to diet and maximally tolerated statin therapy for treatment of adults with familial hypercholesterolemia (FH), with clinical atherosclerotic cardiovascular disease, in those who require additional lowering of plasma LDL-C, or in those who are statin intolerant [1, 9].

As a protein, PCSK9 monoclonal antibody is marginally stable and is easily degraded by numerous pathways. Since this drug is delivered via an auto-injection device every 2 to 4 weeks, patients often have to transport several drug devices home from the hospital. Prevention of deterioration in the drug’s effectiveness can be achieved by proper storage at all times [10, 11]. The manufacturers recommend storage in a refrigerator at 2 °C to 8 °C. Alternatively, it may be kept at room temperature (RT) up to 25 °C in its original carton to protect it from sunlight for a maximum of 30 days. However, the drug’s effectiveness when stored in conditions other than the 2 aforementioned conditions remains unknown. The ambient temperature in tropical countries and the average temperature during summertime in many countries are quite often higher than the recommended ceiling temperature of 25 °C. It is, therefore, recommended to transport the auto-injector devices in a cooler, and storage in a refrigerator at home until use in tropical countries. Despite knowledge of the importance of storing this medication according to the manufacturer’s recommendation, improper storage or transportation could unexpectedly occur. Moreover, it is recommended to discard these relatively high-cost drugs immediately if they were not stored under recommended conditions at all times. The aim of this study was to investigate the effect of various temperatures on the stability of PCSK9 monoclonal antibody that this drug could be exposed to during medication handling and transportation in tropical countries or during summertime in cooler countries.

## Materials and methods

### Study protocol

Two commercially available PCSK9 monoclonal antibodies, including alirocumab 75 mg/ml (Praluent®; Regeneron Pharmaceuticals, Inc., Tarrytown, NY, USA) and evolocumab 140 mg/ml (Repatha®; Amgen, Inc., Thousand Oaks, CA, USA), were used to test the effect of various temperatures on the stability of these drugs under 3 different temperature conditions, including room temperature (RT), cooler device with cold pack, and freeze-thaw. The same batches of PCSK9 monoclonal antibody from each company were used to minimize production-related variation (batch number 1091418 for evolocumab, and batch number 9 W0512 for alirocumab).

**Fig. 1:**
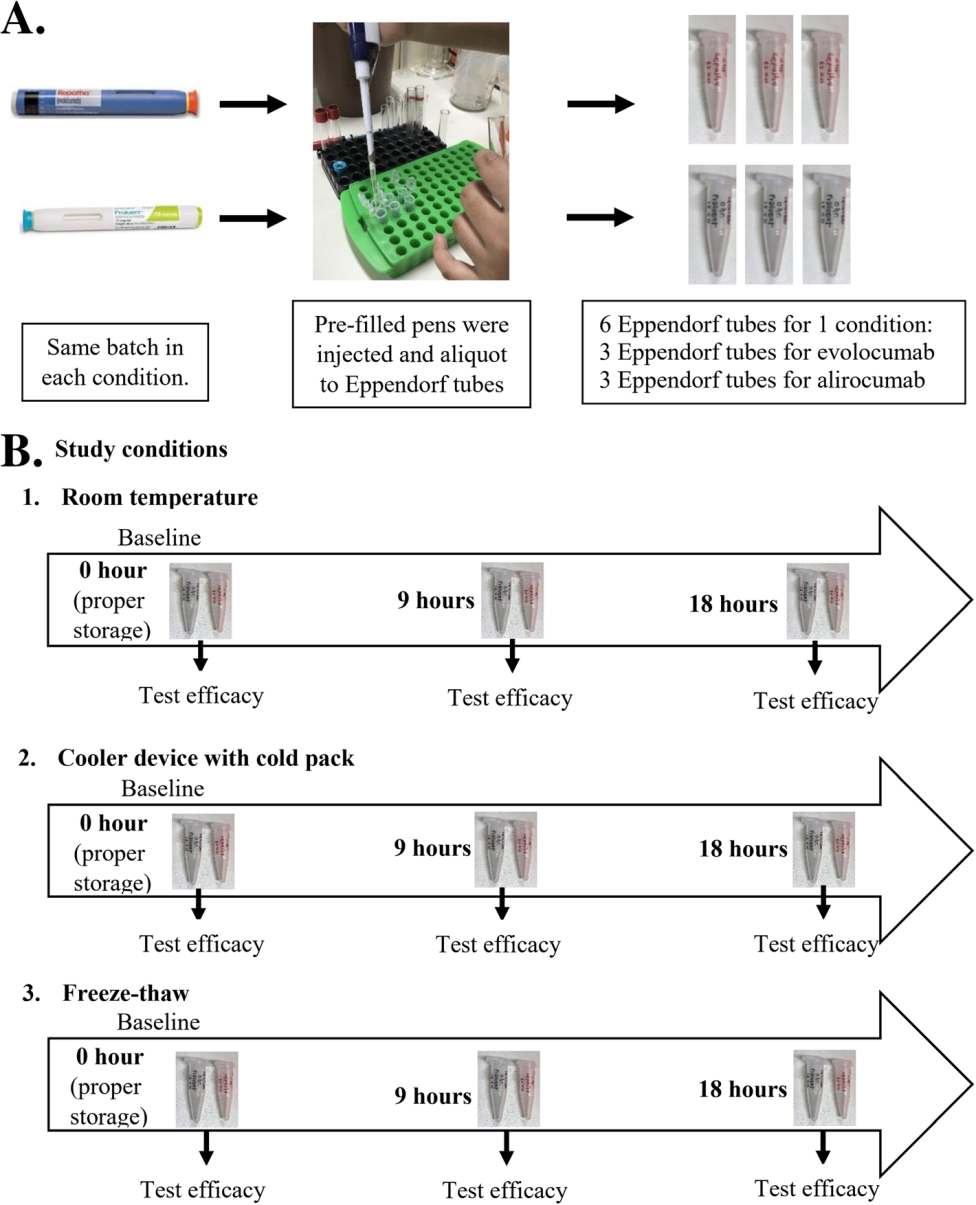
Study design

Drugs were dispensed from pre-filled auto-injector pens, with 160 μl of drug from each pen aliquoted into each of 6 Eppendorf tubes (Fig. 1a). For RT condition, the tested drugs were placed in a closed room without direct sunlight exposure for 9 or 18 h. Temperature recorders (Lascar Electronics Ltd., Wiltshire, UK) were placed together with the drugs to monitor temperature and humidity hourly during the test date. For cooler device with cold pack condition, the study drugs were placed together with a temperature recorder in a standard cooler device (size 14x21x14 cm) with one cold pack (size 8 × 12 cm) for 9 or 18 h. For freeze-thaw condition, the drugs were frozen to -20 °C for 9 or 18 h and then thawed by 27 °C water bath for 2 min (Fig. 1b).

### PCSK-9 quantification

The stability of PCSK9 monoclonal antibody was analyzed using a quantitative enzyme-linked immunosorbent assay (ELISA) method. Serum samples were purified by protein G Agarose (Abcam193258; Abcam, Cambridge, UK) and washing buffer (PBST 20X) before an experiment to separate other IgGs from serum and drug complex. Both study drugs were diluted to half concentration (37.5 mg/mL for alirocumab, and 70 mg/mL for evolocumab). One hundred microliters of each drug was combined with 10 μl of the subject’s plasma and incubated in 37 °C for 20 min. After the binding of free PCSK9 and PCSK9 monoclonal antibody, PCSK9 ELISA kits (Human PCSK9 Simple Step ELISA Kit, ABCAM model ab209884) was used to determine unbound PCSK9 concentration in plasma. The ELISA kit used employed an affinity tag labeled *capture antibody*, and a reporter conjugated detector antibody that immunocaptured the sample analysis in solution. This entire complex (capture antibody/analyte/detector antibody) was in turn immobilized via immunoaffinity of an anti-tag antibody coating the well. The signal intensity was measured at 450 nm by ELISA reader (Ultra reader: EL808; Bio-Tek Instruments, Winooski, VT, USA). Plasma PCSK9 concentration was calculated by comparing sample luminescence to the standard luminescence curve. The PCSK9 concentration in plasma was measured before and after incubation with PCSK9 mAb. The percent inhibition of plasma PCSK9 was calculated using the following formula: (measured PCSK9 level after incubation with drugs – pre-incubation plasma PCSK9 level) / pre-incubation plasma PCSK9 level × 100). Percent inhibition represents the ability of the drug to decrease plasma PCSK9 levels. The stability of the study drugs was inversely related with unbound PCSK9 concentration. High unbound PCSK9 concentration reflects poor drug efficacy. Percent plasma PCSK9 inhibition with proper storage drug was used as baseline. Comparison between percent plasma PCSK9 inhibitions for drugs maintained in each of the 3 study conditions and baseline was calculated and reported as change in percent PCSK9 inhibition from baseline.

This study was conducted in cooperation with the Department of Endocrinology and Metabolism and the Department of Immunology, Faculty of Medicine Siriraj Hospital Mahidol University, Bangkok, Thailand. The study protocol was approved by the Siriraj Institutional Review Board and informed consent was obtained from each patient.

### Sample size calculation

A previous study reported that PCSK9 monoclonal antibody has an ability to reduce plasma LDL-C level by 57.0% [4], and 99.5% of subjects were good responders [12]. The sample size was calculated by using an estimating proportion of one group formula. The relative potency of the study drug to inhibit plasma PCSK9 was 70.0% with an acceptable error of 3.0 and 95% confidence interval. Our calculation showed that plasma samples from 9 subjects were required to test the efficacy of these drugs in the 3 different study conditions.

### Inclusion and exclusion criteria

Patients with dyslipidemia aged over 18 years who visited the endocrine or diabetic clinic at Siriraj Hospital or Phyathai 2 Hospital during September to December 2019 were recruited. The exclusion criteria were patients who were currently being treated with PCSK9 mAb, and patients with history of PCSK9 mAb or immunoglobulin allergy.

### Statistical analysis

The data included demographics (age, gender, hometown), underlying diseases, treatment regimen, and plasma lipid levels. Categorical variables were expressed as percentages, while continuous variables were expressed as mean plus/minus standard deviation (SD) or median (range), as appropriate. Paired *t*-test was used to compare change in percent inhibition of PCSK9 levels at each timepoint of the 3 study conditions. A two-tailed *P*-value of < 0.05 was considered statistically significant. All statistical analyses were performed using SPSS Statistics version 18.0 (SPSS, Inc., Chicago, IL, USA).

## Results

The average age of study subjects was 49.2 ± 18.4 years (range: 19.0–84.0). All 9 study patients were diagnosed with dyslipidemia and were prescribed lipid lowering medications other than PCSK9 monoclonal antibody. Almost 90 % had been taking statin, except one due to statin intolerance. None of them were smokers. Mean fasting LDL-C was 108.1 ± 47.0 mg/dL, and mean total cholesterol was 183.5 ± 53.5 mg/dL (Table 1).

**Table 1:** Baseline demographic and clinical characteristics of study subjects

Characteristics	Patients (***N*** = 9)
Age (yrs), mean ± SD	49.2 ± 18.4
Female gender, n (%)	6 (66.7%)
Diabetes mellitus, n (%)	6 (33.3%)
Hypertension, n (%)	4 (44.4%)
Cardiovascular disease, n (%)	1 (11.1%)
Statin^a^, n (%)	8 (88.8%)
Low-intensity statin	2
Moderate-intensity statin	4
High-intensity statin	2
Ezetimibe, n (%)	2 (22.2%)
Niacin and fenofibrate, n (%)	1 (11.1%)
Alcohol use, n (%)	1 (11.1%)
Laboratory profile, mean ± SD (range)	
Total cholesterol (mg/dL)	183.5 ± 53.5 (127.0–267.0)
LDL-C (mg/dL)	108.1 ± 47.0 (61.8–185.6)
Triglyceride (mg/dL)	96.3 ± 39.5 (47.0–150.0)
HDL-C (mg/dL)	58.0 ± 16.9 (42.0–90.0)
AST (U/L)	26.6 ± 13.0 (14.0–58.0)
ALT (U/L)	22.4 ± 12.7 (10.0–47.0)
BUN (mg/dL)	11.6 ± 2.9 (8.2–16.5)
Creatinine (mg/dL)	0.8 ± 0.2 (0.53–1.14)
Plasma PCSK9 level (ng/ml)	328.41 ± 70.6 (223.4–447.6)

*Abbreviations*: *SD* standard deviation, *LDL-C* low-density lipoprotein cholesterol, *HDL-C* high-density lipoprotein cholesterol, *AST* aspartate transaminase, *ALT* alanine transaminase, *BUN* blood urea nitrogen, ^a^statin: low intensity statin: simvastatin 10 mg/d, pravastatin 20 mg/d, moderate-intensity statin: simvastatin 20 mg/d, atorvastatin 20 mg/d, high-intensity statin: atorvastatin 40 and 80 mg/d

### Heated-unheated condition

Previous study showed that temperature higher than 72 °C for more than 1.5 min resulted in degradation of monoclonal antibodies [13, 14]. Thus, heat stress was performed at 72 °C using a block heater (Wealtec HB-1; Wealtec Corporation, Sparks, NV, USA) for 2 min. Both PCSK9 monoclonal antibodies became clear colorless liquid after heating. Proper storage drugs were kept at 4 °C before incubation with plasma. Plasma PCSK9 levels were measured, and percent PCSK9 inhibition was calculated. As compared with properly stored drug, the mean percent PCSK9 inhibition of heated drugs decreased significantly from 36.4 ± 8.5% to 19.3 ± 10.7% (*P* = 0.004) for alirocumab, and from 37.4 ± 5.4% to 28.1 ± 10.0% for evolocumab (*P* = 0.005).

### Room temperature (RT) condition

**Fig. 2:**
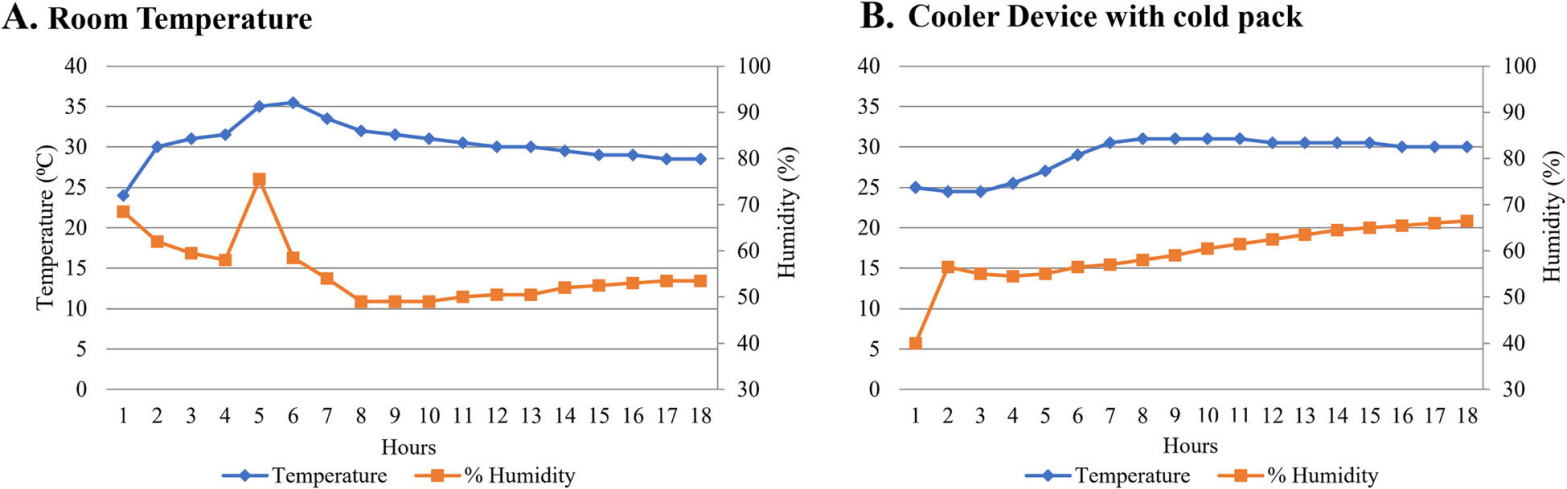
Temperature and humidity by hour during study period: **a** Room temperature condition, **b** Cooler device with cold pack condition

**Fig. 3:**
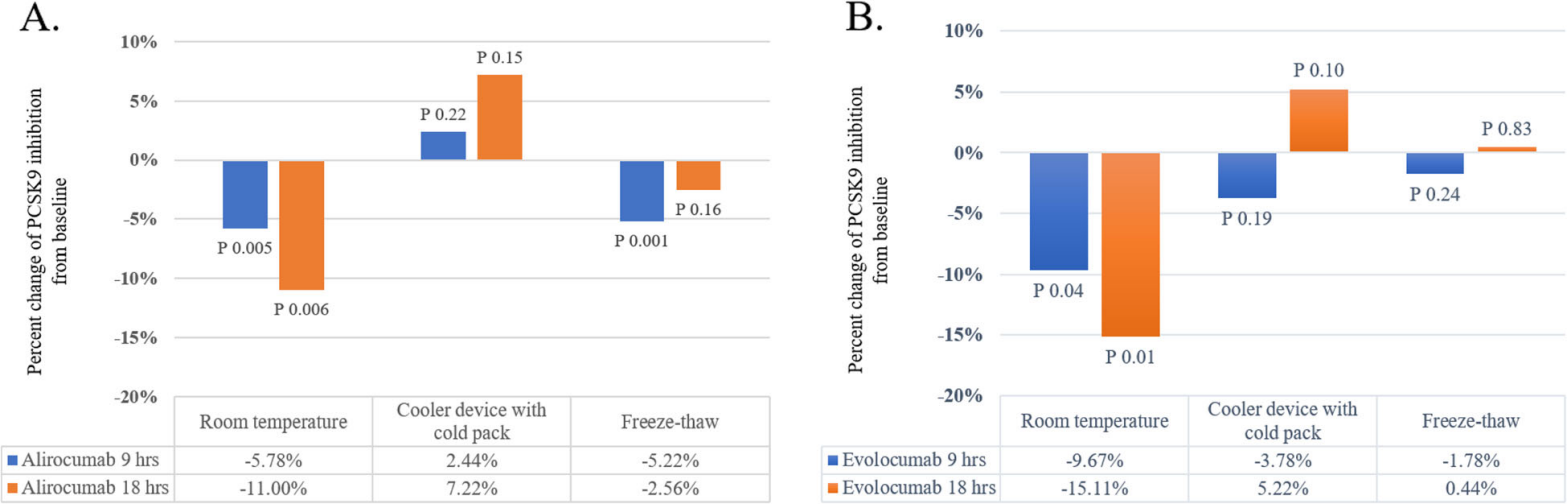
Percent change in inhibition from baseline to 9 and 18 h in each study condition: **a** alirocumab 3.75 mg/ml, **b** evolocumab 7 mg/ml

The average temperature during the study period was 30.4 ± 2.6 °C (range: 24.5–35.3 °C). The duration of temperature higher than 30.0 °C was 9 h. In addition, of the 18 h studied, the duration of temperature higher than 25.0 °C was 17 h (Fig. 2). The manufacturers recommended that these drugs not be stored at a temperature above 25.0 °C. Compared with properly stored drugs, the mean percent inhibition of PCSK9 was significantly decreased from 63.2 ± 15.1% to 57.4 ± 11.5% and 52.2 ± 14.3% after 9 and 18 h, respectively for alirocumab. The mean percent inhibition of PCSK9 was decreased from 65.4 ± 22.9% to 55.8 ± 17.2% and 50.3 ± 18.7% for evolocumab at 9 and 18 h, respectively. There were significant changes in percent PCSK9 inhibition compared to baseline for both study drugs at 9 and 18 h (*P*-value 0.005 and 0.006 for alirocumab, and *P*-value 0.04 and 0.01 for evolocumab at 9 and 18 h, respectively) (Fig. 3).

### Cooler device and cold pack condition

Cooler device with cold pack represents real-life transportation. The average temperature during the 18-h study was 29.0 ± 2.4 °C (range: 25–31.5 °C). The temperature gradually increased from 25.0 °C to RT in 6 h. Later, the temperature remained relatively stable at about 30 °C (Fig. 2). Mean percent inhibition of PCSK9 was 49.1 ± 14.1%, 51.6 ± 13.8%, and 56.3 ± 14.8% for alirocumab, and 53.4 ± 16.3%, 49.7 ± 15.9%, and 58.7 ± 18.1% for evolocumab at 0, 9, and 18 h, respectively. Both study drugs did not show a statistically significant change in percent inhibition of PCSK9 mAb compared to baseline after storage in the cooler device with cold pack for 9 and 18 h (*P*-value 0.22 and 0.15 for alirocumab, and *P*-value 0.19 and 0.10 for evolocumab at 9 and 18 h, respectively) (Fig. 3).

### Freeze-thaw condition

This condition represents unexpected improper storage situation that could occur if the patients store the drugs in the freezer. Mean percent inhibition of PCSK9 was 50.9 ± 7.3%, 45.7 ± 8.6%, and 48.3 ± 10.0% for alirocumab, and 50.7 ± 8.4%, 48.9 ± 7.7%, and 51.1 ± 10.6% for evolocumab at 0, 9, and 18 h, respectively. As compared with properly stored drugs, percent inhibition of PCSK9 mAb dropped significantly after storage of alirocumab in the freezer for 9 h (*P* = 0.001). There was a trend toward a reduction in plasma percent PCSK9 inhibition at other timepoints, but statistically significant difference was not reached.

## Discussion

Alirocumab and evolocumab are indicated in adults with primary hypercholesterolemia or mixed dyslipidemia who require additional lowering of plasma LDL-C despite maximum tolerated dose of statin therapy or statin intolerance [1, 9]. PCSK9 monoclonal antibodies injected subcutaneously every 2 to 4 weeks lower plasma LDL-C level by 50–60% above that attained by statin therapy alone. The manufacturers of these drugs recommend storage of unused PCSK9 monoclonal antibody in the refrigerator at 2–8 °C. Alternatively, these drugs can be kept at RT up to 25 °C for 30 days in the original carton to protect from light [15, 16]. However, RT in a tropical country was usually higher than 25 °C, and by keeping the drugs at RT condition for 9 h or longer resulted in significantly decreased effectiveness of the drug as measured by percent plasma PCSK9 inhibition compared with that of properly stored drugs. Moreover, since the diluted drugs were used in this study, the magnitude of decreased effectiveness of the drugs kept at RT higher than 25 °C could even be greater with patients injecting undiluted drug.

Monoclonal antibodies are immunoglobulins with a precise target. They are proteins that are composed of four chains, two light chains and two heavy chains that are linked together with disulfide bridges. Their quaternary structure is degraded by many different instability mechanisms, including thermal stress. A sufficiently high temperature accelerates various degradation pathways, such as formation of aggregates, fragmentation from peptide bond cleavage, oxidation, and deamidation [13]. More extreme heating also affects monoclonal antibody instability [17]. High temperatures can perturb native protein conformation to a degree sufficient to promote aggregation [18]. In the protocol, heated drugs were used as negative controls in this study. As expected, heating of alirocumab and evolocumab at 72 °C for 2 min significantly decreased percent PCSK9 inhibition of both drugs when compared with the PCSK9 inhibition of properly stored drugs. Therefore, the heated drugs helped to validate the study method.

Since PCSK9 monoclonal antibodies have to be injected every 2 to 4 weeks, several drug devices are normally prescribed and dispensed at each doctor visit, and these patients must transport these devices from the hospital to their residence. Despite emphasis of the manufacturer’s recommendation that PCSK9 monoclonal antibodies be stored at the appropriate temperature at all times, improper storage or transportation can occur. In this study, a possible real-life inappropriate storage or transportation at temperatures that deviated from manufacturer’s recommendation was simulated. The results showed that the efficacy of both tested PCSK9 mAb drugs significantly reduced after storage at RT for 9 h, and the decrease was even more pronounced after storage at RT for 18 h. Heat destroys proteins, and storage of PCSK9 mAb at temperatures higher than 25 °C might deteriorate protein and protein function. Even though this study was conducted during January 2020, which is during the cold season in Thailand, the temperature was higher than 25 °C for 17 of the 18 h on the study day. In addition, the temperature was above 30 °C for 9 of the 18 h (with a maximum temperature of 35.3 °C) on the study day.

In contrast, PCSK9 monoclonal antibodies had acceptable stability and efficacy when stored in a cooler device with a cold pack for up to 18 h. The differences in drug stability between RT and cooler device conditions can be explained by the shorter duration of drugs stored at high temperature, and the lower maximum temperature in the cooler device with a cold pack than the RT condition (max 31.5 °C vs. 35.3 °C). A cooler device with 1 cold pack was able to maintain the temperature under 25 °C for 4 h, after which the temperature gradually increased to RT.

Freezing of monoclonal antibody disturbs protein conformation and leads to aggregate formation during long-term storage. This study demonstrated significantly (*P* < 0.05) decreased function of PCSK9 mAb for alirocumab at 9 h, and non-significantly (*P* > 0.05) reduced function of PCSK9 mAb for evolocumab function at 9 h. This might be the result of the fast-freezing feature of the laboratory freezer used in this study, which is faster than the freezing rate of a freezer in a home refrigerator. Slow freezing of monoclonal antibody was shown to associate with increased protein aggregation [19].

### Study strength and limitations

This study has several important strengths. First, an extensive literature review revealed no previous study that investigated the effect of temperature in a tropical country on the stability of PCSK9 monoclonal antibody. Second, the study design included possible storage and transportation condition that could happen in real-life situation. For example, storage in a cooler device with a cold pack is appropriate for transporting and maintaining the drug for up to 18 h. Third, ELISA method was used to evaluate the stability of PCSK9 monoclonal antibody [20]. Compared with other methods, ELISA is a biological stability study. Residual free PCSK9 is considered an endpoint when investigating the stability of a monoclonal antibody. Quantitative ELISA as a standard method to measure human PCSK9 was developed. Parallelism of residual free PCSK9 within the method was observed across the nine plasma samples tested in this study. This implies that all values of PCSK9 across dilution points were within the targeted acceptance molar ratio (drug:ligand). Fourth and last, in the pre-analytical phase, serum samples were purified with protein G Agarose to separate other IgGs that could interfere with the binding of free PCSK9 and monoclonal antibody. However, this study has some limitations. First, this was an in vitro study because ethical concerns would arise if this investigation were conducted in an in vivo setting. Second, percent inhibition of PCSK9 mAb at baseline in this study was less than previous report [4, 5] because diluted drugs were used. To achieve the best molar ratio in quantitative ELISA test, the study drugs were diluted to 3.75 mg/ml for alirocumab, and 7 mg/ml for evolocumab. However, this study compared the difference in percent inhibition of PCSK9 mAb at each timepoint with the percent inhibition of properly stored PCSK9 mAb used as baseline; therefore, this should not impact the interpretation of the results of this study. Lastly, in the freeze-thaw condition, laboratory freezer was used instead of a household refrigerator. Thus, the temperature was lower in the laboratory freezer than in the freezer of a household refrigerator (− 20 °C vs. -4 °C). Further investigations are needed to ensure generalizability of our findings.

## Conclusions

To achieve the maximum therapeutic effect of PCSK9 monoclonal antibodies, appropriate storage and transportation are essential. It is recommended that all healthcare providers and patients should store PCSK9 monoclonal antibody at a temperature within the temperature range recommended by the manufacturer. However, if inappropriate storage unexpectedly occurs, this study demonstrates that the biological activity of alirocumab and evolocumab remained stable after storage in a cooler device with a cold pack for up to 18 h. In contrast, storage at RT over 30 °C for at least 9 h resulted in significantly decline in PCSK mAb function.

## Supplementary Information

**Additional file 1: Suppl 1.** Percent inhibition of free PCSK9 after storage of PCSK9 monoclonal antibody in heated condition compared to proper storage condition using alirocumab 3.75 mg/ml and evolocumab 7 mg/ml.

## Data Availability

The collection of data for this study is available from the authors upon reasonable request.
