# Supplementary material for: The effect of temperature on the stability of PCSK-9 monoclonal antibody: an experimental study
Source: Lipids Health Dis. 2021 Feb 25;20:21. doi: 10.1186/s12944-021-01447-3 (PMC7905620; doi:10.1186/s12944-021-01447-3)
Supplement: Supplementary file 1 — Additional file 1: Suppl 1. Percent inhibition of free PCSK9 after storage of PCSK9 monoclonal antibody in heated condition compared to proper storage condition using alirocumab 3.75 mg/ml and evolocumab 7 mg/ml. [file 12944_2021_1447_MOESM1_ESM.docx]

**Supplement**

| 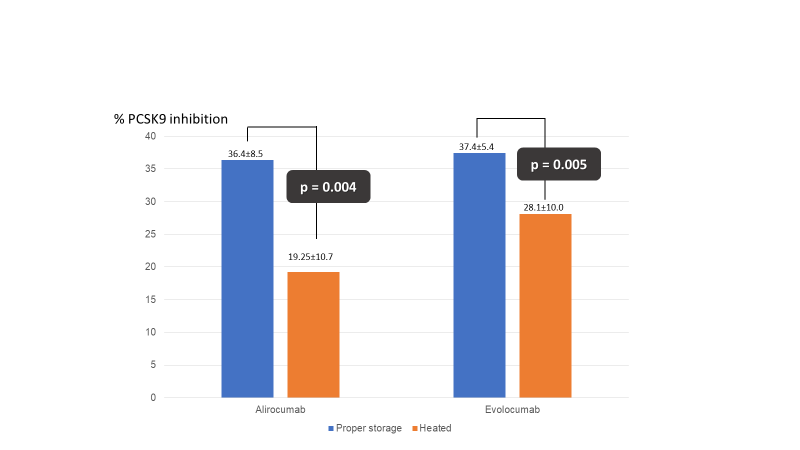 |  |
| --- | --- |

**Suppl 1.** Percent inhibition of free PCSK9 after storage of PCSK9 monoclonal antibody in heated condition compared to proper storage condition using alirocumab 3.75 mg/ml and evolocumab 7 mg/ml.
